# Effects of Music on Attention-Deficit/Hyperactivity Disorder (ADHD) and Potential Application in Serious Video Games: Systematic Review

**DOI:** 10.2196/37742

**Published:** 2023-05-12

**Authors:** Marina Martin-Moratinos, Marcos Bella-Fernández, Hilario Blasco-Fontecilla

**Affiliations:** 1 Department of Psychiatry Puerta de Hierro University Hospital - Majadahonda Majadahonda Spain; 2 Faculty of Medicine Autonoma University of Madrid Madrid Spain; 3 Faculty of Psychology Autonoma University of Madrid Madrid Spain; 4 Department of Psychology Pontifical University of Comillas Madrid Spain; 5 Spain Biomedical Research Networking Center for Mental Health Network Madrid Spain; 6 Ita Mental Health Korian Madrid Spain

**Keywords:** attention-deficit/hyperactivity disorder, music therapy, music, video games, rhythm, timing deficits

## Abstract

**Background:**

Attention-deficit/hyperactivity disorder (ADHD) has a considerable impact on an individual’s daily life. Some difficulties with timing deficits may be associated with deficiencies in attention, reading, language skills, or executive function. Music therapy, either active (playing an instrument) or passive (listening to music) has demonstrated its efficacy in reducing symptomatology in many disorders. Video games may prove to be a useful assessment and treatment tool in compensating for the difficulties with multimodal treatment in ADHD.

**Objective:**

The aim of the study is to (1) analyze the evidence that music is beneficial in reducing the symptomatology of ADHD using systematic review and (2) propose the application of music in video games following music therapy strategies.

**Methods:**

Searches were conducted in PubMed, Embase, PsycINFO, Cochrane, and gray literature (Google Scholar and WorldCat). We used the following search syntax: ((music[Title/Abstract]) or (music therapy[Title/Abstract])) and (attention deficit disorder[MeSH or thesaurus term]).

**Results:**

Of the 70 records identified, 17 provided findings that music can be beneficial in various domains of ADHD. Active music therapy improves hemispheric synchrony, social skills, aggressivity, and impulsivity. Passive music therapy improves academic skills like arithmetic, drawing, and reading comprehension, as well as attention and disruptive behaviors. The effects depend on the music genre, tempo, or task difficulty. Music in video games was generally found to be beneficial for people with ADHD. Music improves immersion and flow while playing video games. Using rhythm may also improve timing skills and immersion in patients with ADHD. Regarding the proposed application of aspects of music to therapeutic video games for ADHD, some paradigms in timing and music therapy were considered in the proposed design of video games.

**Conclusions:**

Improving ADHD treatment through the application of music in video games is proposed.

**Trial Registration:**

PROSPERO CRD42021288226; https://www.crd.york.ac.uk/prospero/display_record.php?RecordID=288226

## Introduction

Attention-deficit/hyperactivity disorder (ADHD) is the most frequent neurodevelopmental disorder with a worldwide prevalence of 5% [1]. Despite its efficacy, multimodal treatment of ADHD (eg, pharmacological, psychological, and psychoeducational modes) is insufficient to fully correct the disorder [2]. Accordingly, ADHD persists in around 65% of cases in adulthood [3] and is complicated by a high rate of comorbidity, accidents, and mortality, among others [4]. Methods based on music therapy, such as musical performance or listening to music, have reportedly reduced symptoms in Parkinson disease [5], brain damage, schizophrenia [6], substance use, posttraumatic stress disorder [7], and neurodevelopmental disorders such as autism spectrum disorder and ADHD [8]. Music therapy is mainly applied in two possible modalities: (1) “active” music therapy when the participant performs music using a musical instrument or voice and (2) “passive” music therapy when the participant listens to music performed by others. Listening to and practicing music activates both hemispheres in sensory, motor, cognitive, language, and emotional areas [9]. Musicians’ brains have better sensorimotor connectivity, greater frequency coherence, and major volume in the basal ganglia, corpus callosum, and cerebellum [10,11]. Music also facilitates changes in our mood and emotional intensity [9], arousal [7], and attention [12]. Furthermore, dopamine, the critical neurotransmitter associated with ADHD, modulates reward circuits associated with music, providing pleasure responses similar to sex, food, or money [13].

Recent neuropsychological models suggest ADHD deficits are implicated in 3 independent pathways: the dorsal frontostriatal pathway involved in cognitive control, the ventral frontostriatal pathway involved in reward processing, and the frontocerebellar pathway related to temporal processing [14]. Temporal processing and auditory cortex morphology could be a biomarker of ADHD, attention-deficit disorder (ADD), and dyslexia, with common patterns in abnormal interhemispheric asynchrony, differentiating each subgroup with 89%-98% accuracy [11,15,16]. Temporal processes seem to normalize with methylphenidate [17], although there is no consensus [18]. People with ADHD have difficulty discriminating seconds and milliseconds in perceptual timing tasks, display worse performance in motor timing and temporal foresight [18], and have difficulty with timing-based rhythm [19]. This impairment of timing skills is involved in the daily life of people with ADHD, for example when planning the consequences of present actions [14], impulsive decision-making, delay aversion, or inappropriate behaviors—such as inattention and motor agitation—in time constraints situations [19]. There is evidence that musical training (especially playing an instrument) can accelerate the development of timing skills and, consequently, the development of the auditory cortex in the long term [11]. Despite this, a recent review of music therapy in neurodevelopmental disorders found only 5 studies of music therapy application in ADHD [8].

Currently, alternative treatments have tried to compensate for the problems associated with multimodal treatment in ADHD. These alternative treatments include neurobiofeedback [20], mindfulness [21], virtual reality [22], and video games [23]. In particular, a number of serious video games have been developed in the last few years to treat symptoms of ADHD, among other disorders. Unlike commercial video games, serious or therapeutic video games are designed for purposes beyond entertainment, usually for education or health uses; in this work, we focus on their application in mental health. Some recent systematic reviews [23,24] found strong evidence of the beneficial effects of serious video games for ADHD. However, none of the studies included in these reviews specifically focused on the effect of music in the efficacy of video game–based treatments. Music and video games in ADHD have mostly been treated separately. Furthermore, both in the clinic and in research, the temporal problems of ADHD have not been sufficiently addressed [18]. Music can be an additional element in the design of treatments for ADHD, especially in therapeutic video games. This review focuses on the possible benefits of music in ADHD and includes a proposal for its possible application in serious video games.

## Methods

**Figure 1:**
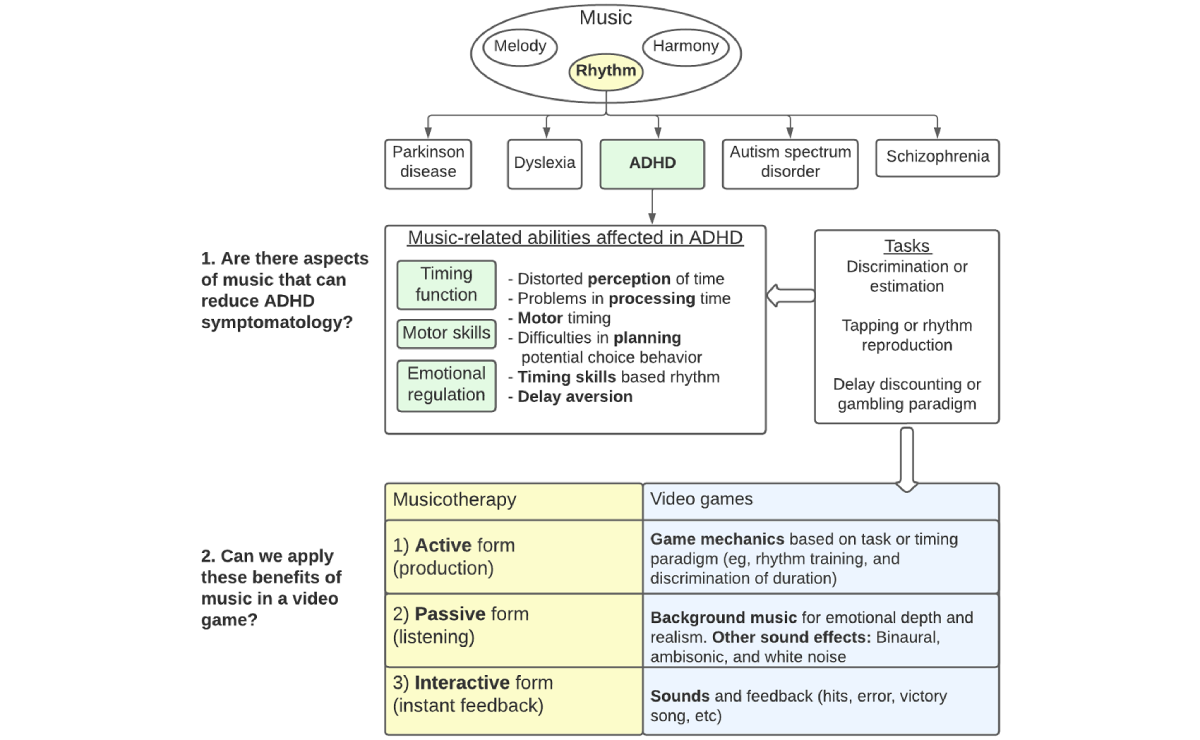
Paper design: music-related abilities affected in ADHD and video game application. ADHD: attention-deficit/hyperactivity disorder.

An initial search was performed using the terms (music[MeSH Terms] and video games[MeSH Terms] and ADHD[Title/Abstract]), with no results. To amplify our search, we then searched for so-called “gray literature.” Gray literature refers to any scientific material not controlled by commercial publishers, such as doctoral and master’s dissertations or technical reports [25]. To do that, a similar search on Google Scholar was performed, and the first 100 entries for eligibility were evaluated. We also performed a search on WorldCat, the largest gray literature database. The study was divided into two sections: (1) a systematic review of symptom reduction in ADHD with the use of music or music therapy and (2) a proposal for the integration of these elements into the design of video games (see Figure 1).

**Figure 2:**
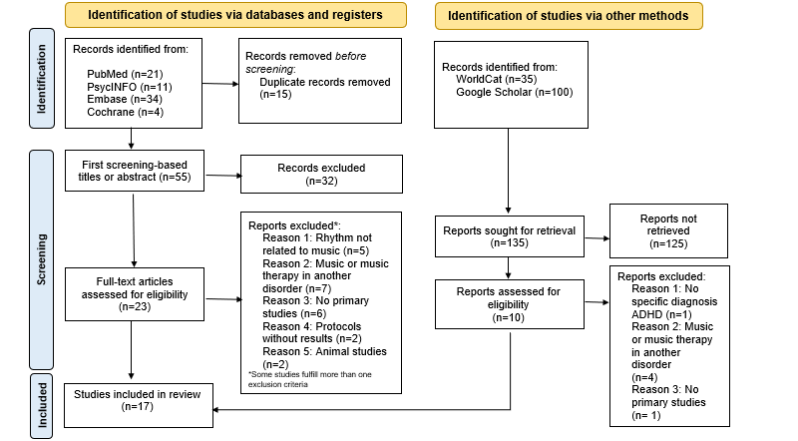
Flowchart of the process following PRISMA statement [27]. PRISMA: Preferred Reporting Items for Systematic Reviews and Meta-analyses.

A PI(E)COS (Populations, Interventions/Exposures, Comparators, Outcomes, Studies) [26] approach was used for the formalization of the research question. The intervention focused only on music treatment, music therapy, or rhythm-based training. The population was children, adolescents, and adults with ADHD. The included studies used the groups themselves (pre-post comparisons) or control groups as comparators. The studies were clinical trials, case-control studies, or pre-post comparative studies. The outcomes were findings related to cognitive, motor, emotional, and social functions. The Preferred Reporting Items for Systematic Reviews and Meta-analyses (PRISMA) guidelines for systematic reviews were followed (see Figure 2).

The inclusion criteria were (1) studies that apply music or music therapy in people with ADHD; (2) randomized clinical trials, prospective comparative studies, and case-control studies; (3) papers published in peer-review journals; and (4) written in English, French, or Spanish. Searches were conducted in English, French, and Spanish in PubMed, Embase, PsycINFO, and Cochrane. We used the following search syntax, adapted for each database: ((music[Title/Abstract]) or (music therapy[Title/Abstract])) and (attention deficit disorder[MeSH or thesaurus term]). The exact syntaxes used for every search and database are described in Multimedia Appendix 1. The search was conducted between October 25, 2021, and May 19, 2022. Two researchers (MMM and MBF) performed the searches. After eliminating duplicates through the Rayyan platform, a first screening was carried out by MMM based on titles and abstracts. Then, a second, full text–based assessment was carried out by MMM and MBF. Disagreements between the 2 evaluators were resolved by consensus. When information was unclear, we contacted authors of the reports to provide further details. MMM was involved in collecting data from the reports. MBF reviewed these data. Data extraction included author, year, sample, musical intervention, outcome measure, experiment conditions, general results, and conclusions. Two researchers (MMM and MBF) assessed the level of evidence of the study. We used a classification with 5 levels of evidence, from high-quality randomized clinical trials (level I) to expert opinions (level V) [27]. This classification method has been adopted by the North American Spine Society and the American Society of Plastic Surgeons. The Principal Investigator, HBF, supervised and corrected the paper. This review is registered in PROSPERO (CRD42021288226), pending approval.

## Results

No results were found for the 3 search terms chosen (music, video games, and ADHD) in the databases, so we searched for gray literature in Google Scholar and WorldCat. The systematic review search was refocused on symptom improvements in ADHD due to music or music therapy.

### Aspects of Music That Can Reduce ADHD Symptomatology

A total of 70 papers were identified from databases of which 15 duplicates were eliminated. Fifty-five papers were evaluated based on titles and abstracts. Of these, 32 reports were excluded due to not being related to the area of our review. Twenty-three full-text papers and 10 gray literature papers were evaluated. A final total of 17 papers were included: 8 from PubMed, 2 from PsycINFO, 3 from Embase, 1 from WorldCat, and 3 from Google Scholar, not counting duplicates (see Table 1). The selected study years range from 1981 to 2020. From them, 6 music-producing studies were found with instruments playing (active music), 10 papers when music was listened to (receptive music), and 1 study in which an interactive metronome was used to work on rhythm (interactive music). The possibility of performing a meta-analysis was discarded due to the heterogeneity in study designs and outcomes. Instead, we extracted one or more statistical outcomes from each study. Results are shown in Table 1 in order to synthesize and integrate them [25,29]. For the outcome integration, we particularly focused on the music modality (active, passive, and interactive) on one side and the ADHD symptomatology treated through music on the other side, grouping the studies for this analysis rather than treating each one of them individually [30].

Effects of active and passive music therapy on people with ADHD are summarized in Textbox 1. Active music therapy was effective in facilitating hemispheric synchrony [11], self-esteem [31], improving social skills [32], and decreasing aggressive behavior [33]. Music therapists perceive music therapy as an effective method in ADHD treatment in combination with other interventions such as medication or psychotherapy [34]. Regarding passive music therapy, we found 4 studies using music as a distractor; the results indicate that music did not produce higher distraction in any case [35-37]. Six studies found that listening to music can help in specific abilities such as arithmetic skills [35], reading comprehension [38], balance performance [36], attention [39], or reduced disruptive behavior [37,40]. In regard to the style of music, we found 2 studies showing reductions in motor activity when children with ADHD were listening to rock music [37,40]. Two other studies used relaxing music [36,38], another one used jazz music [41], and 3 other ones used classical music [39,42,43]. Heart rate variability (HRV) in ADHD was significantly lower when listening to calm music, while HRV was not significantly affected in the control group [38]. However, negative effects were also found by listening to silence and binaural sound. Vorster [44] found that binaural sound reduced attention in people with ADHD, while Zimmermann et al [43] explored the effect of silence on increased arousal and decreased positive mood.

**Table 1:** Aspects of music or music therapy that can reduce ADHD^a^ symptomatology.

Level of evidence			Study		Sample: N, age (years), medication		Musical intervention		Outcome measure		Experiment conditions		General results		Conclusions
**Active form: Producing music**															
	III	Serrallach et al [11]		N=147 (ADHD=37, ADD^b^=36, dyslexic=37, comorbid=15, control=37) Age ADHD: 10.8 (1.9) years; ADD: 11.0 (2.6) years		Musical practice (assessed by IMP=index of cumulative musical practice based on number of years of formal music and hours per week of practicing)		Morphometric, auditory evoked fields and auditory skills. Neuroimaging magneto-encephalography and psychoacoustics		(1) musicians and (2) nonmusicians		Those with ADHD have lower scores in rhythmic (*P*=.005) and melodic (*P*<.05) tasks. Enlarged planum temporale in left auditory cortex (dyslexia, ADHD, and ADD *P*=.005) and to a lesser extent in right auditory cortex (dyslexia: *P*<.05, ADHD: *P*=.005). The Heschl’ gyrus/planum temporale ratios of musicians were higher (1.86±0.9) than nonmusicians (0.85±0.08), especially in the right hemisphere.		Playing a musical instrument for 3 years reduces two-thirds of interhemispheric asynchronies in dyslexia, ADHD, and ADD.	
	IV	Gooding [32]		N=45 children with social deficits. Unspecified how many had ADHD Age: 6-17 years		Drumming, instrument playing, singing, and improvisation		Social skills (Likert scale)		(1) school, (2) residential, and (3) after-school care		Significant improvements (*P*<.05) in social functioning in (1) pre-post self-rating, (2) pre-post rating by researcher, (3) pre-post case manager, (4) pre-post care participants, and (5) behavioral observations.		Music therapy was effective in improving social competence in children and adolescents with social deficits.	
	IV	Rickson [45]		N=13 with ADHD with learning difficulties and comorbid diagnosis Age: 11-16 years Medication		Instructional sessions (playing on percussion instruments and rhythmic activities) and improvisation sessions		Impulsivity (Synchronized Tapping Task) and improve symptomatology (Conners Global Index)		(1) Waitlist control group (n=5), (2) improvisational-instructional group (n=4), and (3) instructional-improvisational group (n=4)		No statistical difference was found in Conners Rating Scales. Musical intervention decreases errors with respect to the control group (*F*=10.419, *df*=2; *P*=.004). No statistically significant difference between instructional or improvisational treatments was found (*P*=.250).		Instructional and improvisational music therapy can help reduce impulsivity.	
	IV	Rickson and Watkins [33]		N=15 with aggressive behavior (n=12 with ADHD) Age: 11-13 years		16 sessions of music therapy (listening, musical instruments, and rhythm-based activities)		Aggressive behavior (Developmental Behavior Checklist)		(1) Group A music therapy (n=6), (2) group B music therapy (n=5), and (3) control group (n=4)		Parent scores show improvement across all subscales for music therapy treatment and waitlist control while teacher results were less consistent.		Music therapy program may help to increase awareness of other people’s feelings.	
	V	Sholeh and Supena [31]		N=22 (5 with ADHD) Age: unspecified		Musical therapy treatment (unspecified)		Observation and interviews		Unspecified		Students with ADHD experienced greater self-acceptance, stronger listening skills, major tendency to complete tasks and make decisions, and positive attitudes.		Music can promote the improvement of participants’ self-esteem.	
	V	Jackson [34]		N=98 music therapists		Music and movement, instrumental improvisation, musical play, and group singing		Improvement in ADHD symptomatology		Survey		The survey results indicate music therapy is perceived as an effective treatment for ADHD in combination with medication (87%) or psychological services (53%). The most proposed goals are behavioral (94%), psychological (89%), and cognitive (69%).		Music therapists perceive music therapy as an effective treatment for ADHD.	
**Passive form: Listening to music**															
	II	Vorster [44]		N=11 with ADHD Mean age: 21.85 years		Different music experiences		Four tasks: (1) Stroop task, (2) sustained attention task, (3) working memory task, and (4) time perception task by functional near-infrared spectroscopy		(1) Binaural beats, (2) classical music, (3) music of preference, and (4) ADHD medication		Listening to music enhanced concentration and performance in people with ADHD. Binaural beats impact negatively sustained attention in comparison to classical music, music of preference, and medication.		ADHD brains respond to musical experience. Attention is affected by musical experience depending upon musical form.	
	III	Abikoff et al [35]		N=40 (20 with ADHD, 20 controls) Age ADHD: 10.08 (1.51) years No medication		Listening to music as a distractor		Academic task performance (arithmetic examinations)		(1) 10 minutes of music, (2) 10 minutes of background speech, and (3) 10 minutes of silence		ADHD scored significantly better in the music condition than in the speech (*P*=.005) or silence (*P*<.05). If they heard the music first, those with ADHD performed better. Control group had similar results under the 3 conditions.		Additional tasks such as music can stimulate children with ADHD to do monotonous tasks, stay longer, and avoid boredom.	
	III	Aydinli et al [36]		N=46 (26 with ADHD, 20 controls) Age: 7-12 years No medication		Listening to relaxing music or white noise as a distractor		Balance performance		(1) Silence, (2) relaxing music, and (3) white noise		Auditory distractors positively affected both groups. White noise was more effective than silence in ADHD (*P*=.001), especially in difficult conditions; relaxing music helped controls more than silence (*P*=.012).		Music or white noise can help better in balance performance more than silence.	
	III	Kiran [39]		N=7 (2 with ADHD, 5 controls) Age: 10-13 years		Playing Tetris while listening to music		Brain waves		(1) Beethoven, (2) Mozart, and (3) no music		Beethoven’s music increased α and β waves in children with ADHD; Mozart’s increased β waves and decreased α waves in the control group.		Listening to classical music such as Beethoven or Mozart can be impactful on children’s attention.	
	III	Klein [42]		N=80 (40 with ADHD, 40 controls) Age: 5-5,6 years		Listening to songs with tempo distorted by compressor (slower and faster)		Repetitive motor responses in musical background conditions. Drawing task		(1) No musical background, (2) classical music with slow tempo, and (3) classical music with fast tempo		Children with ADHD made significantly more errors than the control group under the fast tempo condition (*F*=6.03, *df*=1.77; *P*=.005). The slow tempo condition brought the performance of the children with ADHD closest to the control group. For the unstructured task of free drawing, most children with ADHD performed best under the no-music condition.		For children with ADHD, slow tempo could bring their performance closer to the control group; fast tempo increased the number of errors. Silence was more beneficial in drawing tasks.	
	III	Madjar et al [38]		N=50 (25 ADHD, 25 control) Age ADHD: 12.05 (1.18)		Listening to music		Reading comprehension performance		(1) Without music, (2) calm music without lyrics, (3) calm music with lyrics, and (4) rhythmic music with lyrics		Reading comprehension significantly improved under the music conditions in the ADHD group (*F*_3,135_=6.85; *P*<.001; partial η^2^=0.14) and deteriorated among the control group (*F*_3,72_=4.31; *P*=.005; partial η^2^=0.15). Heart rate variability in ADHD was significantly lower under music condition (*F*_3,72_=3.78; *P*<.05; partial η^2^=0.14).		For children with ADHD, calm music may assist in regulating their autonomous responses and enhance their performance. For controls, listening to music will probably cause a distraction when reading and will debilitate their learning processes.	
	III	Pelham et al [37]		N=67 (41 with ADHD, 26 controls) Age: 8-13 years Medication		Listening to music as a distractor (rock or rap music at 64-74 Hz)		Behavioral intervention performance		(1) No distractor, (2) music, and (3) video		Disruptive behavior of children with ADHD was exacerbated by video condition but not in music condition. Boys in neither group (control and ADHD) were significantly distracted by music. In music condition, 9% ADHD had worse performance, 61% ADHD had no change, and 29% had improved performance.		Listening to music while doing homework may help some children with ADHD more than a silent environment.	
	III	Salmi et al [41]		N=80 (51 with ADHD, 29 controls) Age: 31.02 (8.3) years ADHD without medication during the experiment		Music and white noise as a distractor during film viewing		Altered brain dynamics to function magnetic resonance imaging data		(1) No distractor, (2) jazz music, (3) speech, and (4) white noise		During the music distractor, intersubject correlation group difference was observed in the precuneus and cuneus bilaterally (cluster extent corrected *P*<.05, cluster definition threshold *Z*>2.5).		Desynchronization of the posterior parietal cortex occurred when irrelevant speech or music was presented but not during white noise condition or no distractors condition.	
	III	Zimmermann et al [43]		N=84 (40 with ADHD, 44 controls) Mean age: 30 years		Listening to Mozart’s music for 10 minutes		Subjective arousal (Global Mood-Arousal Scale) and mood (Current Mood Scale)		(1) Mozart’s music and (2) silence		Listening to Mozart’s music decreased negative mood in all groups. In ADHD, a silent condition increased arousal and negative mood.		Silence can influence mood in people with ADHD: increasing arousal and decreasing positive mood.	
	IV	Cripe [40]		N=8 with ADHD Age: 6-8 years Medication		Listening to rock music		Behavior (walk, jump, run, etc) and attention		(1) No music, and (2) rock music (max 58 dB)		Statistically significant reduction (*P*=.005) in the number of motor activities during music period. No significant differences regarding attention span.		Rock music tends to produce a reduction in skeletal muscle tension resulting in reduced motor activity.	
**Interactive form: Music-making with feedback**															
	III	Puyjarinet et al [46]		N=21 with ADHD Age: 7-13 years		Rhythm training for 12 sessions by metronome or music		Sustained, selective, divided attention; inhibition; delay aversion; working memory visuospatial, planification		The training program was divided by age (1) 7-9 and (2) 10-13		After a 12-session rhythm training, children and adolescents with ADHD reduced their impulsivity (Matching Figure Test: W=110; *P*=.005), and visual-spatial working memory was improved (Corsi blocks: W=21; *P*=.005).		Rhythmic training may help improve visual spatial working memory and reduce impulsivity.	

^a^ADHD: attention-deficit/hyperactivity disorder.

^b^ADD: attention-deficit disorder.

**Effects of active and passive music therapy on people with attention-deficit/hyperactivity disorder.**

**Active music therapy**

- Hemispheric synchrony
- Self-esteem
- Social skills
- Aggressive behavior
- Impulsivity

**Passive music therapy**

- Arithmetic skills
- Reading comprehension
- Drawing task (slow tempo)
- Balance performance
- Attention (but decreased when using binaural sounds)
- Disruptive behavior
- Motor activity (rock music)
- Heart rate variability (relaxing music)
- Arousal and mood (negative when using silence)

### Potential Application of Music in Video Game Design

No scientific literature on video games and music applied in ADHD was found in PubMed, Embase, PsycINFO, and Cochrane. Most of the video game studies found do not focus on timing skills or musical abilities [23], and music applied in ADHD was not used in video games but in other contexts (Table 1). Only one video game was found in Google Scholar, “ADDventurous Rhythmical Planet” [47], which combines video games and music for children with ADHD aged 8 to 12 years. This video game works on social skills through rhythm. Music creation through technology has also been explored in other disorders such as autism spectrum disorder and severe intellectual disabilities facilitating social communication skills [48].

Video games are generally effective and well-accepted tools for cognitive skills training in ADHD. The immediate feedback from video games provides direct performance support for individuals with ADHD; they tend to have engagement rates with low numbers of dropouts and increased participation, motivation, and sense of agency [23]. Repetition of tasks through game mechanics is done by increasing difficulty with therapeutic goals [49]. Despite the potential therapeutic impact that specific game mechanics involving rhythmic skills can provide [19], we have not found any video game that considers timing skills in ADHD. However, music video games have been used in some fields of health promotion [50]. In other disorders, namely, Parkinson disease, video games such as “Rhythm Workers” focused on beat perception via finger tapping [49,51]; “GenVirtual” uses a musical game with augmented reality to aid motor and cognitive rehabilitation [52].

Therefore, we focused on applied music therapy methods (active, passive, and interactive) to design specific video games to study timing deficits in ADHD. Audio may improve interactivity in video games in two ways, active and passive: a player causes sounds with his actions (active) and music can modulate the focus and emotional state of the player (passive) [53]. Due to the relevance of timing skills, therapeutic video games may include the training of music in specific tasks (active). For doing so, it is essential to rely on paradigms that have demonstrated validity in training related to timing deficits in ADHD. Based on Noreika et al [18], there are specific tasks for measuring related aspects. Timing deficits could be reduced through training or assessed through motor timing (eg, free tapping, sensorimotor synchronization, or rhythm reproduction task), perceptual timing (eg, with a discrimination task, verbal duration estimation, or a duration production or reproduction task), and temporal foresight (eg, with major difficulty to work it through delay or temporal discounting or gambling paradigms) [18]. These timing tasks can be combined with some ideas for commercial games with specific treatment goals for people with ADHD [50,54].

Regarding the passive way of using music in serious video games, the effect of listening to music in video games is unclear. A study suggests that music reduces the experienced duration of playing with or manipulating time perception [55]. However, this study emphasizes that the effect of music on video games is a complex phenomenon. Immersion and flow state also could depend on the music chosen in the game (whether we like it or not) or could be due to other factors related to game mechanics or enjoyment of the game. An effective sound design can serve to improve the realism and emotional depth of the player experience (eg, terror genre video games) [53]. Overall, music can provide an aesthetic in the game and reduces the possibility of boredom [55]. Moreover, white noise in a bandwidth of 65-80 dB or binaural sound could be incorporated into video games to enhance beneficial effects in ADHD [56]. Other aspects to consider are that effective sound design can facilitate the gamers’ experience (eg, victory songs associated with achievement or sounds indicating error can guide the player in achieving gaming goals). Finally, every player action should receive sound feedback (eg, select from the menu or reach a target, among others).

## Discussion

### Principal Findings

Music is a complex cognitive challenge that apparently has not provided us an evolutionary advantage. However, music provides us great pleasure [13], and brain activation is associated with plenty of complex demands [9,11].

It is a commonly accepted statement that children with ADHD need reduced environmental distraction and silence to perform better. On the contrary, some studies indicate that listening to music before or during a task could improve attentional performance better than silence [35,37,38]. In the case of children with neurotypical development, they also improved their performance with background music [12] except in reading comprehension tasks, where calm music distracted them, while people with ADHD improved their performance [38]. However, the effect of background music on attentional improvement could be modulated by the arousal state of participants, musical elements (eg, tempo, loudness, or the presence or absence of lyrics) [12,42], or difficult task outcome [35,36,43]. In one study [42], the tempo of a song was distorted (faster vs slower), finding performance improvements in slower tempo or silent conditions in children with ADHD, depending on the type of task. Regarding tasks, results suggest that in the case of ADHD, external stimulation with music can help adjust arousal to an optimal level in monotonous tasks. However, when the task is complex, external stimulation may worsen performance [35]. In hard tasks, some studies suggest that white noise may be effective in specific tasks like speech recognition, reading, writing speed, and working memory [36,56]. According to the cognitive-energetic model of ADHD arousal [57] or optimal stimulation theory [58], the distractibility of children with ADHD is a functional attempt to modulate themselves. Silence could generate a major seeking of novelty or stimulation [35] or even provoke distressing mood states during wait times in adults with ADHD [43]. Regarding style of music, the rhythmic and intense beat of rock music stimulate greater brain arousal that overrides environmental distractions [40], while relaxing music decreases HRV, affected in ADHD [38], and decreases negative mood [43].

Regarding active music performance, regularly playing a musical instrument can promote interhemispheric synchronization in people with ADHD, although this higher neural efficiency does not reach the musically trained controls. The choice of musical instrument can help increase practice frequency. Drums or guitars were preferred by people with ADHD, while piano was the preferred choice in children with dyslexia [11]. Moreover, active music can provide significant improvements in social functioning [32] and impulsivity [45]. Another aspect that has not usually been considered is whether it is the musical practice of a solo instrument or of an orchestral instrument which, a priori, could have a beneficial effect on social relations. Furthermore, rhythm gives temporal structure to the music. Following the beat involves sensorimotor integration between predictive (top-down) and reactive (bottom-up) processing where attentional control is involved; regular beat is supported by one’s internal clock, which seems to be faster in ADHD [10]. We tend to move following the rhythm of the music [46], and sounds that follow a pattern can induce stabilization of breathing or movement [59].

As mentioned earlier, people with ADHD have timing deficits, which can range from milliseconds and seconds (eg, perceptual timing and motor timing) to days or years (eg, temporal foresight) [18]. Moreover, patients with dyslexia, ADHD, or ADD have abnormal interhemispheric asynchrony (10-40 ms) of the primary auditory evoked P1 response and oversized left planum temporale as compared with controls [11]. Children with ADHD may also show difficulties in timing-based rhythm [19] or lower scores in rhythmic and melodic tasks [11]. Timing deficits are negatively correlated with impulsivity and delay aversion [60]. Rhythm training may be one more area to integrate into cognitive training in ADHD; some results suggest that after a 12-session rhythm training, children with ADHD reduced impulsivity and improved visual-spatial working memory [61]. A rhythm-based video game for ADHD named “ADDventurous Rhythmical Planet” [47] is associated with improvements in social skills in children aged 8 to 12 years [48]. Training based on rhythm could be integrated into video game mechanics [19]. However, available musical games on the market are not satisfying it because the task in these video games consists only of reacting to visual stimulation while music is presented (eg, games consisting of catching objects at a precise moment or dancing games where players execute movements mimicking a model) without therapeutic goals or with insufficient feedback [54]. Designing specific games that train in timing skills may be especially beneficial in ADHD treatment.

This study has limitations. Few studies were found while searching the databases, so we opted for broadening the search using gray literature from Google Scholar and WorldCat. In addition, the studies were quite heterogeneous, so a meta-analysis could not be carried out. Instead, we qualitatively integrated outcomes. We structured the outcomes considering 2 main variables, music therapy modality (active, passive, and interactive) and reduction in symptomatology, but qualitative reviews are nonetheless more prone to biases in their conclusions [29]. These results should then be interpreted with caution. More specific research is needed to investigate the effects of music on motor and neurodevelopmental disorders such as ADHD. Despite the relevance of timing skills in daily life, it is not sufficiently considered in research and clinical practice [18]. Timing skills could be affected in daily life activities such as predicting the precise moment when a vehicle approaches, particularly in some specific ADHD profiles (because timing deficits may not be present in all of them) [19]. Assessing whether timing skills are affected in people with ADHD may bring us closer to a clearer profile [11,18]. Some authors such as Serrallach et al [11] suggest that auditory cortex patterns could be a differentiating biomarker in ADHD with great relevance in the clinic. The treatment of the 3 pathways mentioned by Sonuga-Barke et al [14] related to executive functions, delay aversion, and timing skills should be considered in order to approach an adequate treatment profile and understand the heterogeneity of ADHD.

### Conclusions

The benefits of music as a part of serious video games for people with ADHD have not been directly assessed. In general terms, this systematic review shows that active (playing music), passive (listening to music), and interactive (music-making with feedback) music therapy are beneficial in reducing ADHD symptomatology and increasing task performance in people with ADHD. Rather than being a distraction, music can help to modulate emotional and cognitive states. Rhythm seems to be a music component particularly benefitted by improved timing perception and regulation. We propose integrating music into the mechanical design of ADHD serious video games, especially related to rhythm-based mechanics.

## Appendix

Multimedia Appendix 1 Search strategies.

## Abbreviations

ADHD: attention-deficit/hyperactivity disorder
HRV: heart rate variability
PI(E)COS: Populations, Interventions/Exposures, Comparators, Outcomes, Studies
PRISMA: Preferred Reporting Items for Systematic Reviews and Meta-analyses
