# Supplementary material for: Effects of Music on Attention-Deficit/Hyperactivity Disorder (ADHD) and Potential Application in Serious Video Games: Systematic Review
Source: J Med Internet Res. 2023 May 12;25:e37742. doi: 10.2196/37742 (PMC10221503; doi:10.2196/37742)
Supplement: Multimedia Appendix 1 [file jmir_v25i1e37742_app1.docx]

**Table S1. Search strategy terms for *“Aspects of music/music therapy that can benefit ADHD symptomatology”.* Results screened in Rayyan platform.**

| Sources | Search strategy | Results |
| --- | --- | --- |
| Pubmed | ((MUSIC[Title/Abstract]) OR (MUSIC THERAPY[Title/Abstract])) AND (ATTENTION DEFICIT DISORDER[MeSH Terms]) | 21 |
| Embase | (music:ab,ti OR 'music therapy':ab,ti) AND 'attention deficit hyperactivity disorder':ab,ti | 34 |
| PsycInfo | (TI music OR AB music OR TI "music therapy" OR AB "music therapy") AND (TI attention deficit hyperactivity disorder) | 11 |
| Cochrane | (("ADHD"):ti OR ("attention deficit disorder"):ti) AND ((MUSIC):ti,ab,kw OR ("music therapy"):ti,ab,kw) | 4 |
| Google Scholar | (“music” OR “music therapy”) AND (“ADHD” OR “attention deficit hyperactivity disorder”) | +100 |
| Worldcat (open access) | (“music” OR “music therapy”) AND (“ADHD” OR “attention deficit hyperactivity disorder”) | 35 |

**Table S2. Search strategy terms for *“Potential application of music in video games design”.* Results screened in Rayyan platform.**

| Sources | Search strategy | Results |
| --- | --- | --- |
| Pubmed | ((MUSIC[Title/Abstract]) OR (MUSIC THERAPY[Title/Abstract])) AND ((video games[Title/Abstract]) OR (videogames)[Title/Abstract]) AND (ATTENTION DEFICIT DISORDER[MeSH Terms]) | 2 |
| Embase | (music:ab,ti OR 'music therapy':ab,ti) AND 'attention deficit hyperactivity disorder':ab,ti AND (videogames:ab,ti OR 'video games':ab,ti) | 2 |
| PsycInfo | (TI music OR AB music OR TI "music therapy" OR AB "music therapy") AND (TI attention deficit hyperactivity disorder) AND (TI “video games” OR TI videogame OR AB “video games” OR AB videogames) | 0 |
| Cochrane | (("ADHD"):ti OR ("attention deficit disorder"):ti) AND ((MUSIC):ti,ab,kw OR ("music therapy"):ti,ab,kw)AND ((videogames):ti,ab,kw OR ("video games"):ti,ab,kw) | 1 |
| Google Scholar | (“music” OR “music therapy”) AND (“ADHD” OR “attention deficit hyperactivity disorder”) AND (“video games” OR “videogames”) | +100 |
| Worldcat (open access) | (“music” OR “music therapy”) AND (“ADHD” OR “attention deficit hyperactivity disorder”) AND (“video games” OR “videogames”) | 86 |
